# Antibody Fragments as Probe in Biosensor Development

**DOI:** 10.3390/s8084669

**Published:** 2008-08-08

**Authors:** Dirk Saerens, Lieven Huang, Kristien Bonroy, Serge Muyldermans

**Affiliations:** 1 Laboratory of Cellular and Molecular Immunology, Vrije Universiteit Brussel, Pleinlaan 2, B-1050 Brussels, Belgium; 2 Department of Molecular and Cellular Interactions, VIB, Brussels, Belgium; 3 Department of Molecular Biology, Technologiepark 927, B-9052 Zwijnaarde, Ghent University, Ghent, Belgium; 4 Department for Molecular Biomedical Research, VIB, Ghent, Belgium; 5 IMEC, NEXT, Kapeldreef 75, B-3001 Leuven, Belgium

**Keywords:** display technology, affinity, stability, immobilization, immunoassay

## Abstract

Today's proteomic analyses are generating increasing numbers of biomarkers, making it essential to possess highly specific probes able to recognize those targets. Antibodies are considered to be the first choice as molecular recognition units due to their target specificity and affinity, which make them excellent probes in biosensor development. However several problems such as difficult directional immobilization, unstable behavior, loss of specificity and steric hindrance, may arise from using these large molecules. Luckily, protein engineering techniques offer designed antibody formats suitable for biomarker analysis. Minimization strategies of antibodies into Fab fragments, scFv or even single-domain antibody fragments like VH, VL or VHHs are reviewed. Not only the size of the probe but also other issues like choice of immobilization tag, type of solid support and probe stability are of critical importance in assay development for biosensing. In this respect, multiple approaches to specifically orient and couple antibody fragments in a generic one-step procedure directly on a biosensor substrate are discussed.

## 1. Antibodies in sensor applications

Over the past few years, multiple protein biomarkers have been suggested as a diagnostic target based on genomic or proteomic studies. Devices such as biosensors that could measure those biomarkers rapidly (e.g. within 10 minutes) and at very low concentrations (e.g. at fg/ml) would be advantageous in diagnostic development. In particular, the capacity of the biosensor to meet challenges such as sensitive detection and low-level quantification of analytes, will undoubtedly put them more in the spotlight^1^. Biosensors are built up of a biological target-recognition element that is connected to a transduction element using a suitable interface layer. Binding events occurring at this functionalized interface layer are translated by the transducer into a comprehensive read-out^2^. These biosensors provide a rapid, convenient, low cost alternative to conventional analytical methods such as HPLC, ELISA, 2-D gel electrophoresis or mass-spectrometry, for detecting or assaying a biomarker.

One particular category of biosensors is the antibody-based biosensor or immunosensor. This type of biosensor relies on the ability of an immobilized antibody (Ab) to recognize its associated target, known as antigen (Ag). For biosensor development these Ab-based probes should meet very high standards such as high specificity in a very complex medium, well-characterized binding properties, high stability and the possibility of large-scale production preferably at low cost^3^. Another important aspect in biosensor design is the quality of the interface layer between probe and transduction element since it will also determine both the sensitivity and the specificity of the biosensor. Here several problems may arise, as proteins and Abs in general are chemically and structurally complex and heterogeneous. This makes them often unpredictable regarding their interactions with the biosensor substrate^4^. Therefore, it is difficult to define a general protein detection and immobilization strategy^5^.

Immunoassays based on polyclonal (pAb) and monoclonal (mAb) antibodies have been around for more than 30 years and are still among the most important diagnostic tools widely used in clinical and research areas^6^. The pAbs can easily be generated, but batch-related differences, varying affinity and poly-specificity (i.e. reactivity with more than one target) can create serious problems, certainly when used as a probe in biosensors^7^. In contrast, a mAb can be selected to be more specific for a unique epitope present on the protein of interest and/or its variant(s). In addition, any particular mAb can - in principle - be obtained reproducibly in unlimited quantities and its target-affinity can at least be determined. Their identification is amenable to a high-throughput mode by immunizing animals with antigen mixtures followed by automated screening so that large numbers of additional binders per annum are within reach. Moreover, many mAbs are already used as affinity reagents for identification, validation, quantification, localization, functional analysis and ablation of proteins^8^.

Nowadays, these Abs are proposed as prime candidates to be used as probes in biosensors. Despite some successes, a reasonable fraction of the mAbs selected for specific analyte recognition fails to function properly in the biosensor setup due to unpredictable conformation changes on surfaces, or unwanted reactivities mediated by their Fc part. Therefore Abs have previously been minimized into different Ab formats and optimized for affinity and/or stability to improve development of a robust Ab-based probe for biosensor applications^9^. In addition, it has become obvious that immobilization engineering is a mandatory step in the development^10^. In many cases loss of biological activity upon immobilization of Abs is noticeable. One reason might be the random orientation of the Abs on sensor surfaces whereby optimal Ag binding is prohibited compared to soluble Abs^11^.

## 2. Available antibody fragment formats

**Figure 1.**
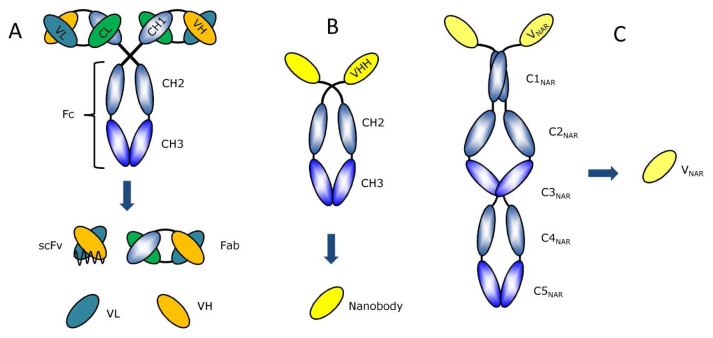
Ab fragments from conventional **(A)**, Heavy-chain **(B)** and cartilaginous fish **(C)**.

Since the introduction of recombinant Ab engineering, the size of mAbs has been minimized and adapted into different formats suitable for the envisaged application^8^. The well-established smaller engineered format of a mAb is the Fab fragment containing the complete Light-chain (VL and CL domain) and the first half of the Heavy-chain, the Fd (VH and CH1 domain) (Figure 1A). The Fab encompasses the Ag-binding domain without the effector function fragment, the Fc part. Even smaller fragments can be designed from the Fab fragment, e.g. an Fv and the single-chain Fv (scFv) fragment^12^.

With a small flexible polypeptide linker between VH and VL domain, the scFv fragment is generally more stable than the Fv fragment, which leads to higher functionality^13^. Unfortunately, this synthetic linker causes dimerization and aggregation of the scFv, subsequently fuelling many investigations into more stable Ag-binding Ab fragments^14^, e.g. the scFab^13^. The smallest possible Ag-binding Ab fragment from a mAb is made up of just one variable domain, e.g. the VH or VL^14^. Unfortunately these fragments have a pronounced tendency to form aggregates as these isolated single-domains often expose large hydrophobic regions to the solvent.

Antibodies have dogmatically been described as being composed invariably of two identical Heavy-chains and two identical Light-chains. The species of the *Camelidae* (i.e. *Camelus dromedarius, C. bactrianus, Lama glama, L. guanoco, L. alpaca and L. vicugna*) however produce a surprising exception to this paradigm. Their serum contains a considerable fraction of Heavy-chain Abs (HCAbs), that lack the Light-chain^15^ (Figure 1B). The Heavy-chain within the HCAbs is composed of three instead of four globular domains. The two constant domains are highly homologous to the CH2-CH3, Fc domains of classical Abs^16^. Remarkably, the domain corresponding to the CH1 domain of classical Abs is missing in HCAbs. Hence, the Ag-binding fragment of a classical Ab, the Fab, is reduced to a single variable domain in the HCAb. This variable domain referred to as VHH^17^ or Nanobody^18^ is adapted to become functional in Ag-binding in absence of a variable Light-chain domain (VL)^19^. It has been repeatedly demonstrated that the VHH, cloned and expressed in bacteria, is a strictly monomeric, single-domain Ag-binding entity^20^. Even more recently, an alternative natural single domain antibody format was discovered in cartilaginous fish, e.g. sharks. A new and probably ancestral immunoglobulin isotype termed “novel antigen receptor” (Ig-NAR) was described^21^. Similar to HCAbs in camelids, this new isotype is a Heavy-chain homodimer. The Ig-NAR is a disulfide bonded homodimer of two identical H-chains that lacks light chains. Each H-chain contains one variable domain (V-NAR) and five constant domains (Figure 1C).

The reduced size, improved solubility and higher stability of the camelid Heavy-chain and shark Ig-NAR antibody fragments are of special interest for biotechnological and medical applications, including biosensors^22^.

## 3. Generation of antibody fragments libraries

Recombinant Ab technologies to engineer mAbs into smaller Ab fragments, although very promising, may cause loss of affinity, increased tendency to aggregate, increased temperature sensitivity, and low yield of protein functional in Ag recognition. Moreover the isolation and subsequent purification of mAbs is a very costly and time-consuming process. These issues can be circumvented by selecting Ag-binding Ab fragments from libraries using *in vitro* screening technologies and bacterial expression of the selected clone. This construction of highly diverse expression libraries of Ag-binding Ab fragments based on combinatorial principles is the first key technology en route to obtain optimal Ab-based probes. An Ab fragment library is usually derived from a single scaffold such as Fab, scFv or VH. Essentially, variability is generated at several regions of the Ag-binding moiety in many different ways; from the random combination of VH and VL domains, to the introduction of variability into the antibody scaffold using synthetic^23; 24^ or semisynthetic^25; 26^ approaches. Several methods were already optimized and resulted in the construction of large scFv libraries^27; 28; 29^. Such hyperdiversified Ab fragment libraries enabled the selection of Ab fragments specific to virtually any target. Besides these synthetic libraries, Ab fragments can be selected from a camelid non-immune library^30^ or immune libraries against a wide variety of antigens^18; 31; 32; 33^. Subsequent isolation of Ag-specific Ab fragments from these libraries can be performed via different screening techniques.

## 4. Selection of antigen-specific antibody fragments

In order to isolate highly potent Ab-based probes from these large libraries, so-called display technologies are the second key technology to identify probes. Display technologies physically link the probes' genotype with its phenotype, and allow very efficient handling of large expression libraries (sometimes encompassing > 10^10^ individual clones). Various forms of display technologies such as phage display^34; 35; 36^, ribosome display^37; 38; 39; 40^ or mRNA display^41^ libraries have been reported. Ribosomal display has the advantage that it does not require bacterial host cells, and thus there is nearly no limit in extension of library complexity. Here genotype and phenotype are linked through ribosomal complexes, consisting of mRNA lacking a stopcodon, ribosome and encoded protein that are used for selection. However due to the high technological demands of ribosome display, widespread application of this technology has been hampered.

The most robust of these *in vitro* selection procedures - and by far the most widely used - is phage display. Phage display has been utilized for isolating recombinant Ab fragments. After construction of an Ab combinatorial library, Ag-specific recombinant Ab fragments can be easily isolated by bio-panning of the phage library displaying Ab fragments fused with viral coat protein III against antigen proteins, antigen-expressing live cells, or fixed cells^36^. Several steps in Ab phage display may be improved by: (i) increasing the size of the library to enlarge the chances to select for high affinity binders within the repertoire, (ii) adapting the bio-panning procedure for isolation of Ab fragments reactive with immunological minor epitopes^42^, (iii) enhancing the expression level and stability of the selected Ab fragments and (iv) engineering of the expression phagemid cloning vector^43^.

Combining the Ab fragment libraries with powerful phage display has led to a multitude of generated Ab fragments. Although these various technologies allow the isolation of highly specific antibody fragments, these fragments do not necessarily meet the functional standards required for successful employment in a biosensor format. These problems can be overcome by use of optimized scaffolds^44^ or stress driven selections (e.g. temperature^45^ or chemical denaturing^32^). Once a suitable Ab fragment has been selected to bind a diagnostically relevant epitope, further engineering can be performed to increase antigen affinity, probe stability or immobilization potential. Different approaches to further improve the Ab properties towards ideal biosensor probes are described below.

## 5. Affinity engineering

High-affinity is a prerequisite for the development of simple and highly sensitive biosensors. Sometimes the Ab fragments selected via display technologies fail to meet the required kinetic-affinity parameters of target association/dissociation to develop an optimal sensor assay. Ideally, the k_on_ value (i.e. the kinetic association rate) needs to be above 10^5^ M^-1^ s^-1^ for rapid assay results (less than 15 minutes). The k_off_ value (i.e. kinetic dissociation rate) seems to be less critical, and values from 10^-3^ s^-1^ are appropriate for acceptable target release. Panning of immune libraries usually yields Ab fragments that bind with nanomolar affinity (K_D_=k_off_/k_on_) to their cognate target. However, binders retrieved after panning of (semi-) synthetic libraries do not routinely reach such low K_D_ values. The improvement of affinity of an Ag-Ab interaction, although challenging, can be tremendous beneficial to develop a sensitive biosensor. Several techniques such as random mutagenesis, direct evolution, ribosome display, etc. can be included to optimize the Ab fragments towards a more suitable k_on_ or k_off_ value. Affinity maturation via a combination of molecular evolution and high-throughput methods (e.g. via ribosome display) is preferred. This combination has resulted in the isolation of specific antibody fragments from naïve libraries, with sufficient affinity for analyte biosensing applications^46^.

## 6. Stability engineering

An overlooked parameter of Ab fragments for immunosensor development is the intrinsic stability. Not only is long-term storage and re-usability of Ab chips important from a practical point of view, but also stability during manufacturing it is a critical factor. Therefore high demands are placed on the functional stability of the probes for biosensors.

The Fv and scFv fragments engineered from mAbs or selected from combinatorial libraries do not always reach the required stability treshold^47; 48^. Upon construction of Fv and scFv fragments, the variable domains are removed from their natural Fab context, where they are associated with the constant domains of the Light-chain (CL) and Heavy-chain (CH1). Despite mutual stabilization of the domains in a scFv, most scFv derived from mAbs show poor to moderate stability without additional engineering^12^. Many research groups have solved the problem of the limited stability of the Fv Ab fragment via different routes. Both VL and VH domains can be fused into many alternative formats, besides the conventional scFv format. In a first format the CH1 and CL domain of the Fab fragment are replaced with a heterodimeric coiled coil, resulting in a helix-stabilized Fv fragment (hsFv)^49^. In a second format, the disulfide-stabilized Fv fragment (dsFv), a cysteine residue is introduced into the conserved framework regions of both the VH and VL at positions compatible with the formation of an interdomain disulfide linkage. This dsFv can bind antigen with identical affinity, and proved to be substantially more resistant to heat or urea treatment than the scFv^50^. Another format to stabilize the scFv is obtained by introducing a disulfide bond into the scFv which results in a ds-scFv, combining the stability of the disulfide form with the expression advantages of the scFv^51^. The reformatting of the scFv into a Fab fragment is an alternative strategy that leads towards an improved functional stability of the Ab fragment. Such approach might - in some cases - replace the additional maturation steps, when the affinity and stability are close to the limit of tolerance for a successful biosensor assay^52^. However, it is rather preferred to minimize the dimensions of the antibody-based probes to get a maximal amount of probe onto the sensor surface, than enlarging the probe into a Fab.

Besides applying optimized formats, the variable domains of suboptimal stability can be engineered for improved robustness and folding efficiency while preserving their Ag-binding specificity and affinity. This can be performed either by introducing a limited number of point mutations^53^ or by grafting their Ag-specificity onto variable domains with frameworks of superior stability. The grafting technique, originally utilized to humanize Abs, involves loop grafting of the CDR-loops of less-stable Ab fragments onto a highly stable framework, resulting in a reshaped Ab fragment with the Ag specificity of the donor Ab and the stability of the acceptor framework^25^. Grafting onto scFv was shown on multiple occasions^54; 55^. In case of camelid Heavy-chain Ab fragments similar results were obtained, i.e. a universal loop acceptor VHH-scaffold was identified and revealed to be able to harbor loops from different VHHs. This allows stabilization of Nanobodies for employment as probes in biosensors or microarrays^56^. Recently, a novel method for increasing the intrinsic stability of Ab fragments was proposed. By inserting a disulfide bond in the hydrophobic core of any variable Ab domain, the chemical and thermal stability could be increased^57; 58^.

The above mentioned techniques are used when the Ab-based probe is already available and stability improvement is required. To increase the level of throughput in Ab-based probe generation, combinatorial libraries can be used in conjunction with stress-guided selection techniques. This resulted already in several general strategies for probe generation^59; 60; 61^.

The Ab intrinsic stability is important during probe regeneration. Biosensor applications involving multiple detection cycles are critically dependent on the feasibility to fully regenerate the probe, i.e. the complete removal of captured analyte from the probe in between two detection cycles. Such regenerations might create an additional difficulty when the cleaning conditions destroy the activity of the probe. It becomes even more difficult for probe-analyte complexes with high affinity constants. These will require extreme conditions to disrupt their interaction. Backmann et al. ^47^ reported on a label free immunosensor using single-chain antibody fragments where repeated regeneration with low-pH buffer resulted in loss of binding activity. This indicated that the covalent immobilized scFv fragments might undergo unfolding and denaturation upon harsh regeneration. Since intrinsic stability of the probe has been shown to be an important parameter for optimal biosensor design^62^, problems with regeneration can be circumvented by selecting Ab-based probes with high intrinsic stability.

## 7. Immobilization of antibody fragments

**Figure 2.**
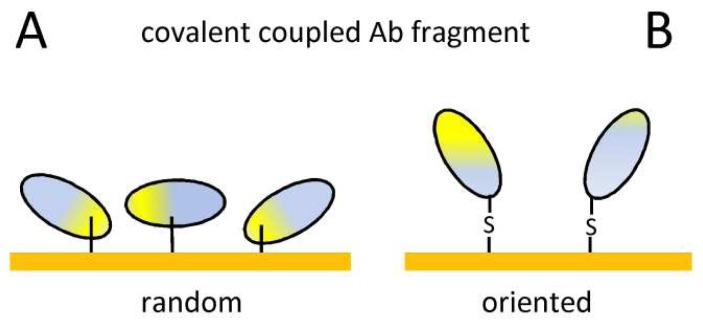
Ab fragment immobilization via random **(A)** or oriented **(B)** covalent coupling.

The attachment of active recognition molecules at high-density to the transducer surface is one of the most critical steps in biosensor development. Proper strategies for Ab immobilization will mainly be determined by the solid sensing substrate and the properties of the interface layer. The most suitable method of Ab immobilization therefore varies with the type of biosensor that needs to be coated with the desired probe. The full-sized Ab has estimated molecular dimensions of 15 × 7 × 3.5 nm^10^, whereas the smallest Ab fragments, i.e. single-domain Ab fragments, are only 4 × 2.5 × 3.5 nm. Moreover, the Ab-Ag interaction is also affected by the nature of the interface layer between the immobilized Ab and the sensor surface^63^. Ideally, the probe should specifically recognize and bind its antigen at the lowest possible concentration. Since the biosensor surfaces are in the μm scale, the smaller the Ab fragment, the more probes can be immobilized onto the surface, resulting in an enhanced detection sensitivity^64^. Unfortunately it has been observed regularly that proteins, and Abs in particular, may lose (part of) their biological activity when immobilized on a surface. This can be attributed to a combination of two factors: change in conformation upon cross-linking and/or unfavorable orientation of the probes^65^ (Figure 2A).

Physical adsorption is the simplest process of protein binding, although rather uncontrollable. It occurs through hydrophilic, hydrophobic or both types of interactions between Abs and the sensor surface. Random orientation of the absorbed Abs and close proximity between adsorptive surface and the Ag-binding site could impede the detection (Table 1).

Covalent attachment of Abs on chemically-activated sensor surfaces is the most common method for Ab immobilization. Numerous chemical coupling reagents (e.g. glutaraldehyde, carbodiimide or succinimide ester) to cross-link mainly carboxylated functional groups have been tested to immobilize Abs onto various solid surfaces. Most commonly used covalent immobilization approaches couple the Abs randomly via their free amino-groups to the chemically-activated sensor surface. Such covalent binding of proteins to a biosensor surface represents a rational and robust approach (Table 1).

However, numerous studies over the past 40 years have shown that the physical adsorption or the covalent attachment procedures of antibodies onto solid supports increase the probability of denaturation or conformational change (Table 1). After surface immobilization, mAbs and recombinant Ab fragments exhibit non-uniform kinetic and thermodynamic properties with the respective Ag. A general solution is to uniformly orient the probes and increase the accessibility of their Ag-binding domains^66^ (Figure 2B).

Overall, the directional immobilization processes share several advantages. Usually, the Ag-binding domains of the probe are better accessible to the analyte when the surface-coupling site within the probe is at a distant position from the Ag-capturing site. Within the Ab population, the Ag-binding kinetics upon covalent immobilization remains more uniform and this can affect the biosensor sensitivity positively^67^. This was emphasized by Bonroy et al. ^68^ when they showed for a particular Ab-Ag pair that the optimized fragmentation protocol in combination with an oriented immobilization of Fab fragments on mixed self-assembled monolayers (SAMs) led to a more than two-fold increase of the Ag binding signals compared to randomly covalent immobilized full-length Abs.

There are different approaches to obtain oriented immobilization of the probes. A first simple immobilization strategy involves the direct chemical coupling of the probe onto the transducer, e.g. a non-coated gold surface and available –SH groups on the probe^69^. Another methods uses a variety of chemical reactions to attach the probe via the –SH group to the gold substrate that is coated with a dextran layer^70^ or sensor or with self-assembled monolayers (SAMs) of thiols^2^ (Figure 2B). Ab fragments such as a Fab fragment, could be immobilized via the native thiol group more easily after reduction of the Fab_2_ fragment^71^. Even an engineered C-terminal cysteine residue at the light chain constant domain of a scFv fragment could be successfully applied to attach the probe via a heterobifunctional linker onto a gold surface^72^.

**Figure 3.**
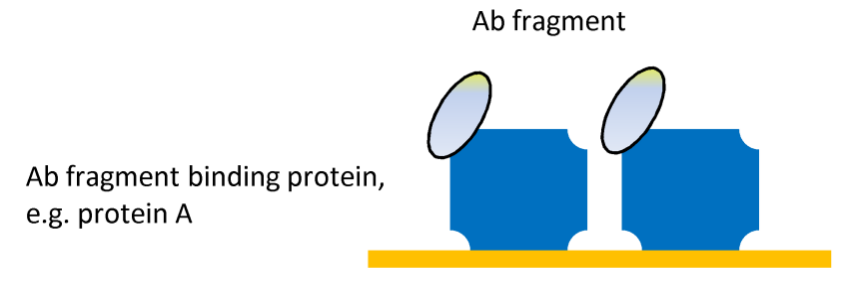
Ab fragment immobilization via intermediate layer.

As an alternative to direct chemical coupling, oriented immobilization can also be achieved indirectly, via an intermediate layer (the so-called ‘immobilization layer’), which is added between the gold thin-film and the immobilized Ab. An elegant example is by utilizing natural Ab binding proteins such as protein A or G for increasing biosensor sensitivity and specificity^73^. Ab-binding proteins such as protein A and G have been extensively employed to capture Abs on biosensor surfaces with their Ag-binding site maximally exposed to the solution and thus remaining fully functional^4^ (Figure 3).

**Figure 4.**
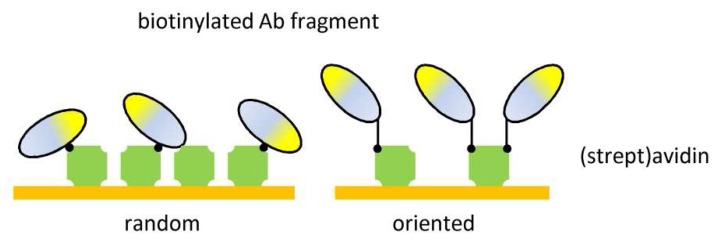
Ab fragment immobilization via biotin-(strept)avidin interaction.

Another method for orienting Abs specifically onto surfaces consists of attachment of biotinylated Abs onto a (strept)avidin-modified surface (Figure 4). Ab fragments can be biotinylated by random or oriented labeling procedures. Random biotinylated scFvs can be coated onto surfaces at much higher densities than most commonly used Abs, improving the biosensor sensitivity and specificity^74^. Site-directed biotinylation of Abs at their hinge region preferentially at the sulfhydryl groups between CH1 and CL domains was developed to immobilize Abs in an oriented manner via biotin-streptavidin linkage. These site-directed biotinylated Abs showed consistently enhanced detection capabilities compared to random biotinylated Ab preparations^75^. Site-specific biotinylation can also be obtained via a novel method for generation of yeast-secreted, *in vivo* biotinylated recombinant antibodies, or biobodies^76^. The camelid single-domain antibody fragments can also be biotinylated *in vivo* at a site-specific lysine residue within a designed tag and can thereafter be captured at high density in a directed orientation on a (strept)avidin coated biosensor substrate^62^.

**Figure 5.**
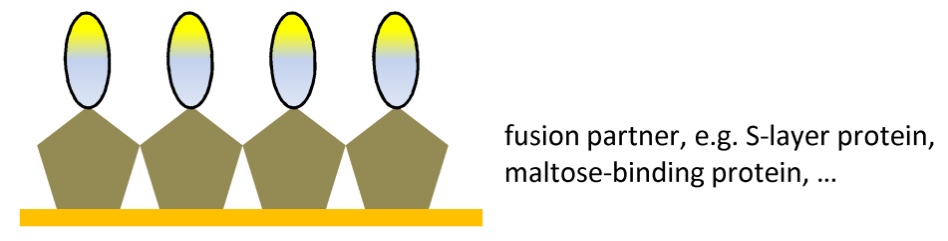
Ab fragment immobilization via fusion partner.

Recently Ab fragments were genetically fused to a range of proteins and subsequently immobilized onto the sensor surface. Fusion of the Ab fragments to a universal immobilization domain will provide identical orientation of all molecules (Figure 5). Pleschberger et al.^77^ succeeded in fusing a bacterial S-layer protein to a Nanobody. This fusion protein retained the ability to self-assemble onto solid surface into a square lattice structures with the Nanobody pointing outwards from the protein lattice surface into the solution. The monomolecular protein lattice could be exploited as a sensing layer in a biosensor setup. A similar approach was used for scFvs, whereby the fusion scFv with pIII surface protein allowed a more sensitive detection in their biosensor set-up^78^. Other chimeric proteins can be made via fusion of an Ab fragment with proteins such as beta-galactosidase, maltose-binding protein, calmodulin-binding protein, chitin-binding domain, cellulose-binding domain^79^ or glutathione-S-transferase.

**Figure 6.**
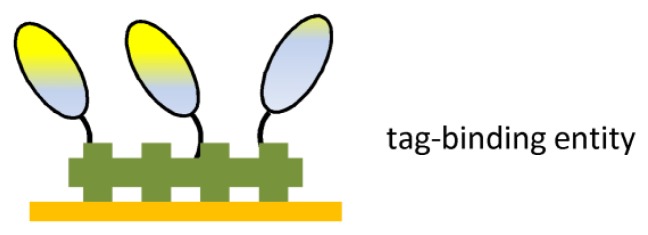
Ab fragment immobilization via affinity tag.

Since most recombinant Ab fragments are expressed with an affinity tag in order to facilitate purification, this tag is also proposed for immobilization onto a sensor surface (Figure 6). His-tag fused Ab fragments could be non-covalently immobilized onto Ni^2+^ or gold surfaces. Several scFv fragments were engineered to contain two histidines within the linker peptide used to join the scFv heavy and light chains^80^. These scFvs bound to the surface in a proper orientation, retained antigen-binding affinity, and could be coupled at high surface concentrations. By replacing the standard single His_6_-tag with a double-His_6_-tag on human recombinant scFvs, the binding onto Ni^2+^-nitrilotriacetic acid-coated substrates was significantly enhanced^81^. These improved binding characteristics enabled non-purified probes as present in crude expression supernatants to be directly applied thereby eliminating the need for any time-consuming pre-purification step(s) prior to the probe immobilization.

**Figure 7.**
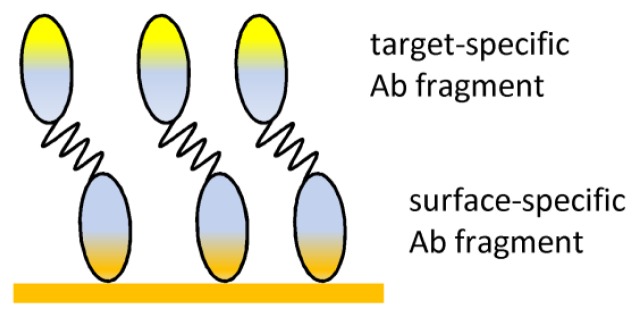
Ab fragment immobilization via bispecific construct containing a Ab fragment specific for the surface.

Moreover Abs with a high affinity for certain types of materials could be advantageous in biomaterial science and be used in an approach to immobilize probes on a biosensor device. Special selected or constructed Ab fragments with high affinity for certain materials can be used, e.g. an Ab fragment that binds to polyhydroxybutyrate, a biodegradable polymer that is often used as biomaterial, was generated by a phage display system. This Ab fragment could be used in bispecific constructs for site-directed oriented immobilization of Ab fragments on this biomaterial^82^ (Figure 7). In addition Ab fragments with binding affinity and specificity for non-biological inorganic material surfaces (e.g. gold) were generated by grafting material-binding peptides into loops of the complementarity determining regions of Abs^83^.

## 8. Examples of antibody fragments in biosensor development

Various studies on the use of antibody fragments as probes for biosensors were reported. For example Mechaly et al. ^84^ developed a scFv fragment for the efficient and specific detection of *B. anthracis* spores, and demonstrated its successful application in detection platforms like ELISA, IFA and FCM. Hu et al. ^85^ optimized a domoic acid-binding scFv antibody fragment and functionally immobilized it on a mesoporous silicate support for biosensor application development. Deng et al. ^86^ isolated scFv fragments against *Clostridium difficile* toxin B. This scFv could detect toxin B to a minimum of 10 ng per well. These examples reveal how the recombinant antibody technology might assist in the rapid and effective development of next generation immunodiagnostic reagents. Huang et al. ^64^ developed a human prostate-specific antigen (PSA) biosensor based on a camelid single-domain antibody fragments. Via covalent immobilization of the VHH onto mixed SAMs and an optimized sandwich assay, clinically relevant concentration of PSA could be measured. In related research, different VHH constructs were immobilized onto commercial and custom-built sensor surfaces by metal chelation, biotin-streptavidin interactions or covalent coupling^62^. For the first time, the intrinsic stability was presented as an important probe design factor, as higher intrinsic stability offers higher resistance to harsh environments. Finally, Carlsson et al. ^87^ used large-scale recombinant scFv antibody microarrays for the first time to classify metastatic breast cancer versus healthy controls, based on differential protein expression profiling of whole serum samples. The miniature assay set-up provided pM range sensitivities and > 95% specificity.

## 9. Concluding remarks

A biosensor is envisaged as a compact, portable device which is (i) very useful in remote or developing regions without easy access to sophisticated laboratory facilities, (ii) amenable to mass production and (iii) minimized for development into a handheld, point-of-care-device^88^. Many biomarkers have been originally proposed as a direct result of −omics investigations. For their subsequent validation, it is essential to possess over highly specific probes that can detect those potential candidates. Antibodies are the natural molecules that fulfill the role of specific reporter molecules in vertebrates. In the post-genomic era and with high-throughput techniques available, the goal is to discriminate between all individual proteins from the proteome including their splice variants and post-translationally modified derivatives. Aided by advances in generation, selection and engineering of antibody-based recognition units, biosensors provide tools for improved diagnostics and biomarker discovery through detection of high- as well as low-abundant analytes even in complex, non-fractionated proteomes in conjunction with usage of small amounts of samples and reagents.

## Figures and Tables

**Table 1.** Advantages and disadvantages of different Ab fragment immobilization methods.

Immobilization method	Advantages	Disadvantages
Adsorption	Minimal manipulation	Random orientation
	No Ab modification	Ab denaturation
	Mostly high immobilization level	Non-specific protein binding
		Leakage of Ab from surface
Covalent coupling	Stable immobilization	Random orientation
	Commercially available surfaces	Ab modification, possible denaturation
Ab fragment tag	Oriented immobilization	Surface stability
	Mild incubation	
Ab-binding proteins	Oriented immobilization	Surface stability
	No Ab modification	
	Mild incubation	
Ab fragment fusions	Oriented immobilization	Compatibility between Ab fragment and
	Surface stability	fusion partner
